# Manybody interferometry of quantum fluids

**DOI:** 10.1126/sciadv.ado1069

**Published:** 2024-07-19

**Authors:** Gabrielle Roberts, Andrei Vrajitoarea, Brendan Saxberg, Margaret G. Panetta, Jonathan Simon, David I. Schuster

**Affiliations:** ^1^ Department of Physics, University of Chicago, Chicago, IL, USA.; ^2^ Center for Quantum Information Physics, Department of Physics, New York University, New York, NY, USA.; ^3^ James Franck Institute, University of Chicago, Chicago, IL, USA.; ^4^ Pritzker School of Molecular Engineering, University of Chicago, Chicago, IL, USA.; ^5^ Department of Physics, Stanford University, Stanford, CA, USA.; ^6^ Department of Applied Physics, Stanford University, Stanford, CA, USA.

## Abstract

Characterizing strongly correlated matter is an increasingly central challenge in quantum science, where structure is often obscured by massive entanglement. It is becoming clear that in the quantum regime, state preparation and characterization should not be treated separately—entangling the two processes provides a quantum advantage in information extraction. Here, we present an approach that we term “manybody Ramsey interferometry” that combines adiabatic state preparation and Ramsey spectroscopy: Leveraging our recently developed one-to-one mapping between computational-basis states and manybody eigenstates, we prepare a superposition of manybody eigenstates controlled by the state of an ancilla qubit, allow the superposition to evolve relative phase, and then reverse the preparation protocol to disentangle the ancilla while localizing phase information back into it. Ancilla tomography then extracts information about the manybody eigenstates, the associated excitation spectrum, and thermodynamic observables. This work illustrates the potential for using quantum computers to efficiently probe quantum matter.

## Teaser

Ancilla-controlled preparation of quantum fluids enables efficient measurement of energy spectra and thermodynamic observables.

## INTRODUCTION

Advances in controllable quantum science platforms have opened the possibility of creating synthetic quantum materials, in which the physical laws governing the material are built to order in the laboratory (*1*–*4*). Such experiments enable time- and space-resolved probes (*5*–*7*) of quantum dynamics inaccessible in solid-state matter, as well as explorations of extreme parameter regimes (*8*–*12*). As the community has become increasingly adept at leveraging the flexibility of synthetic matter platforms to realize arbitrary physical laws, we now face the challenge of capitalizing on this same flexibility for preparing and characterizing quantum manybody states.

In electronic materials, preparing low-entropy equilibrium states relies upon refrigeration: harnessing the coupling of the material to a low-temperature reservoir that can absorb its entropy. By contrast, synthetic material platforms are known for their coherent, low-dissipation evolution, and hence their lack of reservoir coupling. State preparation has thus relied upon the development of new approaches based on engineered reservoirs (*1*, *13*–*17*) and adiabatic evolution (*12*, *18*, *19*), elucidating, among other things, microscopic aspects of quantum thermodynamics (*20*, *21*) and the importance of symmetry breaking (*22*), respectively.

Even once a manybody state is prepared, characterizing it presents unique challenges. The intuitively simplest but technically most demanding characterization approach is state tomography, where *n*-body correlations are measured in complementary bases, allowing complete reconstruction of the system density matrix (*23*). This approach has the advantage that all information about the state is extracted, and the disadvantage that the required statistics (and thus measurement time) scale exponentially with system size. If specific rather than complete information about the state is desired, more carefully crafted protocols have been shown to relax measurement requirements: Expansion imaging measures single-particle coherence (*24*); noise correlations measure two-body ordering (*25*); in situ density probes equation of state (*26*); Bragg spectroscopy is sensitive to density (and spin) waves (*27*); particle-resolved readout accesses higher-order correlations (*5*, *8*, *28*–*30*); parity oscillations are clear signatures of Greenberger–Horne–Zeilinger states (*31*); single-qubit tomography probes global entanglement (*8*, *32*); and shadow tomography (*33*) provides an efficient way to extract observables from few measurements.

It has nonetheless become apparent that treating state preparation and state characterization as independent does not fully leverage quantum advantage—approaches that entangle the two tasks via an ancilla can be vastly more performant: Proposals and experiments to quantify scrambling (*34*–*36*) and verify manybody localization (*37*) rely upon out-of-time-order correlators that compare manybody states to which a specific operator is applied either before or after coherent evolution. This is achieved by entangling the time at which the operator is applied with the state of an ancilla and subsequently performing tomography on the ancilla. Similarly, Loschmidt echoes directly measure the impact of perturbations via state overlap measurements following evolution under two similar Hamiltonians (*38*, *39*). Sensitivity and dynamic range enhancements in sensing (*40*) can be achieved by sandwiching ancilla-conditioned dynamics between quantum Fourier transforms. Entangling initial states with an ancilla and applying ancilla-conditioned evolution can further probe anyon braiding phase (*41*) and system spectrum (*42*, *43*).

Here, we introduce manybody Ramsey interferometry as a direct probe of thermodynamic observables: We entangle which manybody state we prepare in a Bose-Hubbard circuit with the state of an ancilla qubit, allow the superposition to evolve, disentangle from the ancilla, and perform ancilla tomography to learn about the manybody states. We rely upon our recently demonstrated reversible one-to-one mapping of computational states onto manybody states (*32*) to achieve the ancilla/manybody state entanglement. Because we entangle and then disentangle the ancilla from the manybody system, we localize the sought-after information in a single qubit for efficient, high signal-to-noise readout, rather than extracting it from a many-qubit state space (*42*–*45*).

In the following section, we introduce our circuit platform and manybody Ramsey protocol. We demonstrate the protocol and use manybody Ramsey to probe adiabaticity of state preparation. Finally, we use manybody Ramsey to directly measure thermodynamic observables of a strongly interacting quantum fluid by studying superpositions of (i) particle number and (ii) system size.

## RESULTS

### The platform

**Fig. 1.**
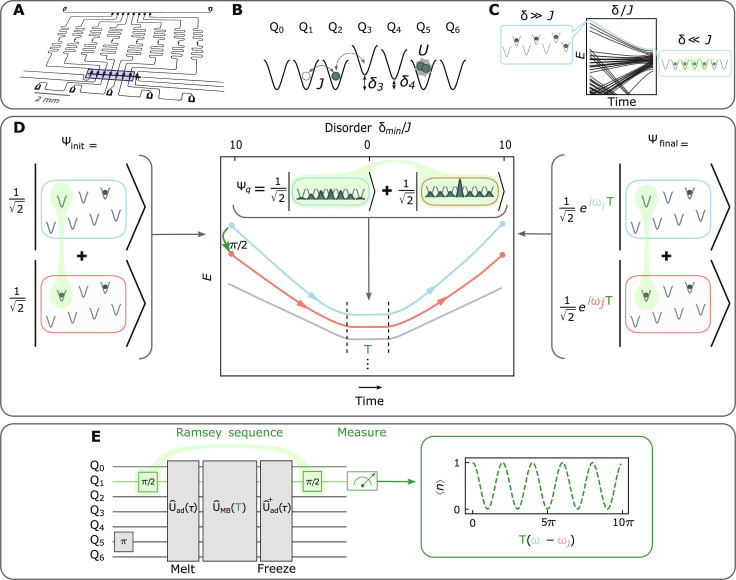
Preparing and interfering manybody states. The quantum system probed in this work consists of a chain of seven capacitively coupled superconducting transmon qubits (*59*) [blue in (**A**)] connected to site-resolved readout resonators (meandering traces) and flux control (bottom traces). (**B**) The system is well described by the Bose-Hubbard model: particles (microwave photons) coherently tunnel between lattice sites (qubits) at a rate *J*, with on-site interactions *U* ≫ *J* arising from the transmon anharmonicity (*1*, *17*, *46*). Real-time flux tuning provides control of lattice site energies (δ*_i_* for site *i*), allowing the deterministic manipulation of disorder that we leverage to build highly entangled states. (**C**) Starting in a highly disordered lattice, we initialize the system in a chosen energy *N*-particle eigenstate by applying π pulses to *N* empty sites (left) and adiabatically removing disorder to convert these states into eigenstates of the quantum fluid (right) (*32*). (**D**) To interfere superpositions of such states, we replace one of the assembly π pulses with a $\frac{\pi }{2}$ pulse on the qubit with a green highlight in the figure (referred to as the ancilla qubit in the main text), producing a superposition of two (red/blue) eigenstates. Adiabatically removing disorder produces a superposition of two manybody fluid states; coherently evolving for a time *T* allows the eigenstates to accumulate a relative phase proportional to their energy difference; ramping back to the disordered configuration (right) relocalizes the phase difference into the single qubit that started in a superposition; a final $\frac{\pi }{2}$ pulse on this qubit maps the phase information onto qubit occupancy for measurement. (**E**) Reinterpretation of the full manybody Ramsey sequence as a set of gates on the qubits comprising the lattice, resulting in an interference fringe versus evolution time *T*.

The properties of our synthetic quantum material platform are accurately captured by a one-dimensional (1D) Bose-Hubbard model (see Fig. 1B), describing bosonic particle tunneling between lattice sites at rate *J*, in the presence of onsite interactions of energy *U*

$$\begin{array}{c} H_{\text{BH}}\left(t\right)/\hbar =J\sum_{\langle i,j\rangle }a_{i}^{†}a_{j}+\frac{U}{2}\sum_{i}‍n_{i}\left(n_{i}-1\right)\\ +\sum_{i}‍\left[\omega_{\text{lat}}+\delta_{i}\left(t\right)\right]n_{i} \end{array}$$ (1)

Our Hubbard lattice is realized in a quantum circuit (*1*, *17*, *46*): Sites are implemented as transmon qubits, particles as microwave photon excitations of the qubits, tunneling (*J*) as capacitive coupling between the qubits (Fig. 1, A and B), and onsite interactions (*U*) as transmon anharmonicity. Lattice site energies (qubit frequencies) can be individually and dynamically tuned using flux bias lines (see Table 1). For this work, *J*/2π = −9 MHz, *U*/2π = −240 MHz, and ω_lat_/2π ≈ 5 GHz. The tuning range of our qubits extends from $\frac{\omega_{\text{qb}}}{2\pi }∼3\text{to}6$ GHz. The photon lifetime *T*_1_ ≈ 40 μs is much longer than the timescale of the manybody dynamics (see Table 1 for details).

**Table 1.** System parameters.

Qubit	1	2	3	4	5	6	7
*U*_lattice_/2π (MHz)	−236	−235	−209	−234	−236	−231	−225
*J*_*i*,*i*+1_/2π (MHz)	−9.62	−9.58	−9.63	−9.74	−9.76	−9.63	–
*T*_1_ (μs)	14.6	35.5	57.7	28.4	60.3	54.7	40.0
*T*_2_^*^ (μs)	0.85	0.64	1.31	0.77	3.57	0.84	1.4

We recently demonstrated adiabatic preparation of photonic fluids by leveraging real-time (≪ tunneling time) control of lattice disorder (*32*). This protocol begins with lattice sites tuned apart in energy by more than the tunneling *J*. In this configuration, the many-particle eigenstates are localized into product states over individual sites such that any eigenstate may be prepared via site-resolved microwave π pulses that inject individual photons. By next adiabatically removing the lattice disorder, we smoothly convert the localized eigenstates of the disordered system into the corresponding highly entangled eigenstates of the disorder-free system (Fig. 1C). The combination of the one-to-one mapping and the ease of state preparation in the disordered (staggered) system renders it straightforward to prepare any eigenstate of the ordered system provided sufficient coherence time to ensure adiabaticity in the disorder-removal ramp.

We now harness this precise eigenstate preparation to explore controlled interference of many-particle quantum states. Our approach can be understood in analogy to traditional Ramsey spectroscopy of a single qubit (*47*) with states |0⟩ and |1⟩: In this simpler case, a system prepared in |0⟩ is driven into an equal superposition of |0⟩ and |1⟩ with a $\frac{\pi }{2}$ pulse, and after an evolution time *T*, the phase accrued between |0⟩ and |1⟩ is read out with a second phase-coherent $\frac{\pi }{2}$ pulse: The resulting population difference between |0⟩ and |1⟩ states oscillates (versus evolution time *T*) at a frequency set by the energy difference between the states.

To extend the protocol to measurement of the phase difference between two manybody states, we take the single-qubit Ramsey sequence above and sandwich the delay time *T* between qubit-conditioned assembly and disassembly of the manybody states. We call this approach “manybody Ramsey interferometry” to connect with previous work exploring non-adiabatic evolution of manybody systems (*36*, *44*, *45*). This procedure maps the phase accrued between the manybody states entirely onto the single qubit, avoiding any reduction in contrast due to residual entanglement, at measurement time, with the manybody system. The enabling ingredient for this protocol is qubit-conditioned manybody state preparation, which we implement via our disorder-assisted adiabatic assembly techniques (*32*).

An example of the full protocol is shown in Fig. 1D. In the presence of disorder, we prepare a superposition of energy states of the *N* = 1 and *N* = 2 particle manifolds $|\Psi_{i}\rangle =\frac{1}{\sqrt{2}}(|0000010\rangle +|0100010\rangle )=|0\rangle ⊗\frac{|0\rangle +|1\rangle }{\sqrt{2}}⊗|00010\rangle$. The key to this protocol is that in the presence of disorder the superposition of the two manybody states is realized as a superposition of a single control qubit, realized with a $\frac{\pi }{2}$ pulse. As we adiabatically remove the disorder, the localized states melt into corresponding eigenstates of the quantum fluid. During the subsequent hold time *T*, these states will accrue a relative phase proportional to their energy difference, (ω*_i_* − ω*_j_*)*T*. Finally, to relocalize the information back into the control qubit, we adiabatically reintroduce lattice disorder, producing the final state: $|\Psi_{f}\rangle =\frac{1}{\sqrt{2}}\left(|0000010\rangle +e^{i\left(\omega_{i}-\omega_{j}\right)T}|0100010\rangle \right)=|0\rangle ⊗\frac{|0\rangle +e^{i\left(\omega_{i}-\omega_{j}\right)T}|1\rangle }{\sqrt{2}}⊗|00010\rangle$. The phase accrued between the manybody states has now been written entirely into the control qubit. We extract that phase information (and thus the manybody energy difference) with a final $\frac{\pi }{2}$ pulse on the control qubit and a population measurement in the |0〉,|1〉 basis. The complete pulse sequence is illustrated in Fig. 1E.

### Demonstration of the protocol

**Fig. 2.**
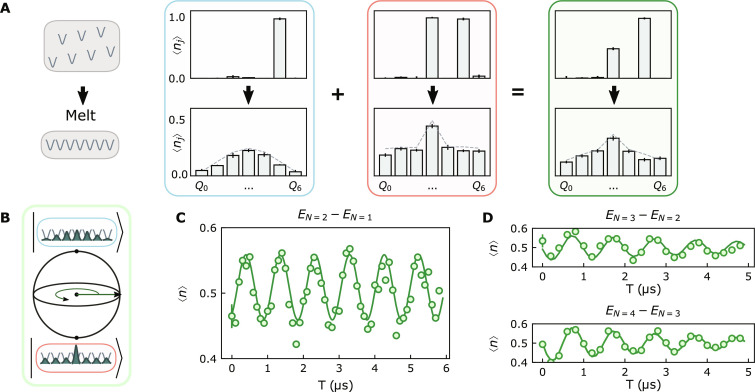
Benchmarking the manybody Ramsey protocol. (**A**) To explore this protocol in the laboratory, we deterministically inject particles into a disordered lattice and remove the disorder (left), before imaging the resulting density distribution. When we inject precisely one photon (upper blue panel), adiabatic disorder removal produces the lowest-momentum particle-in-a-box state (lower blue panel); injecting two photons (upper red panel) produces the lowest-energy two-body state after disorder is removed (lower red panel). If we deterministically inject the first particle with a π pulse but $\frac{\pi }{2}$ pulse the second photon, we should produce the manybody superposition state, and we observe the average of the two density distributions (green panels). (**B**) To demonstrate that this average density distribution corresponds to the macroscopic superposition of manybody states, we allow the superposition state to evolve on the Bloch sphere before adiabatically mapping the manybody superposition back onto a single qubit, where it can be read out via a second $\frac{\pi }{2}$ pulse. (**C**) The resulting Ramsey fringe (versus hold time *T* in the manybody superposition state) evolves with a frequency given by the energy difference between the manybody states minus the frequency of the local oscillator from which the $\frac{\pi }{2}$ pulses are derived, exhibiting contrast over several microseconds limited by the single-qubit *T*_2_ (see the Supplementary Materials). In (**D**), we demonstrate the applicability of the approach to larger systems by applying it to superpositions of *N* = 2, 3 and *N* = 3, 4 particle fluids; the increased decay reflects the faster dephasing of states with more particles. Fits to data in (C) and (D) are plotted as solid green lines; the frequency deviation from numerics are 400 kHz, 2 kHz, and 2 MHz, respectively. Representative error bars (on first data point of each plot) reflect the SEM.

We benchmark our manybody Ramsey protocol by studying the superposition of one- and two-photon fluid ground states in our Hubbard circuit. In Fig. 2A, we prepare these states separately (red and blue boxes) by π-pulsing localized particles into the disordered lattice and then adiabatically removing the disorder, finding good agreement of the measured in situ density profiles with a parameter-free Tonks gas model (*48*, *49*) (see the Supplementary Materials, section C). When the second particle is instead injected with a $\frac{\pi }{2}$ pulse, we create the desired superposition state, with a density profile reflecting the average of the two participating eigenstates (green box).

To measure the energy difference between these states, we must interfere them. We achieve this by replacing the in situ density measurement with a coherent evolution time *T*, allowing the states to accrue a relative phase (Fig. 2B), followed by adiabatically reintroducing the disorder to relocalize the phase information into a single lattice site (qubit) and finally applying a $\frac{\pi }{2}$ pulse to interfere the states and read out the encoded phase in the occupation basis. The resulting sinusoidal Ramsey fringe (occupation versus *T*) is shown in Fig. 2C, with contrast limited in this experiment by qubit dephasing (see Table 1). Both dephasing and dissipation lead to reduction in fringe contrast without changing fringe frequency, while non-adiabatic ramping results in additional frequency components (discussed further in the following sections). The fringe frequency of 10 MHz is translated down (for clarity) from the actual energy difference of 5.317 GHz via a *T*-dependent phase offset of the second $\frac{\pi }{2}$ pulse (see the Supplementary Materials, section A). Similar experiments enable single-qubit measurement of energy differences of 2/3 and 3/4 particle superposition states (Fig. 2D), with minimal contrast reduction and only a small drop in coherence time. We are thus prepared to apply the protocol to exploration of manybody physics.

### Probing the excitation spectrum

**Fig. 3.**
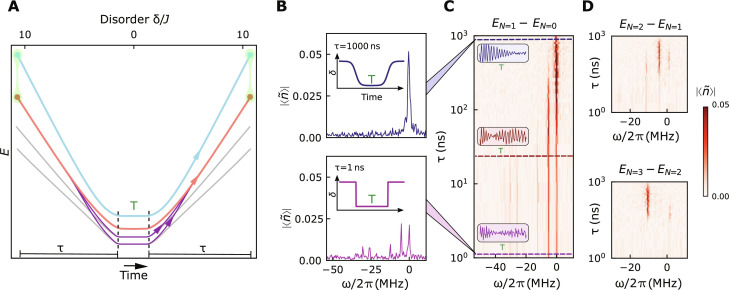
Spectroscopic signatures of adiabaticity. The manybody Ramsey protocol relies critically on the ability to adiabatically map localized states into and out of highly entangled states (in a ramp time τ). (**A**) Ramping too quickly leads to diabatic excitations (purple) into other manybody states that do not interfere with the states (red/blue) in the prepared superposition (green) and thus reduce Ramsey fringe contrast. With some probability, however, these diabatic excitations are diabatically de-excited back into the initial state (red) during the backwards ramp; because these excitations evolve at different frequencies (corresponding to their energies) during the hold time *T*, they produce Ramsey fringes at other Fourier frequencies. (**B**) For the slowest ramp (τ = 1 μs), there are no diabatic excitations, producing a single Fourier feature in the Ramsey interference between *N* = 0 and *N* = 1 eigenstates. (**C**) For the fastest ramp (τ = 1 ns), the many diabatic excitations are reflected in additional frequencies in the Ramsey fringe beyond the dominant feature in the slow ramp. As the ramp time τ is varied over three decades, frequency components furthest from the dominant feature disappear first, with the low-offset-frequency features disappearing only for the slowest ramps, consistent with excitation rates controlled by the energy gaps of the fluid. Insets depict the time-domain Ramsey fringes for slow, intermediate, and fast ramps (top to bottom). (**D**) Repeating these experiments with superpositions of *N* = 1,2 and *N* = 2,3 particles demonstrates that while the proliferation of manybody states makes resolving diabatic excitations challenging, the dominant feature nonetheless appears for the slowest ramps.

Our manybody Ramsey protocol relies upon our ability to adiabatically assemble and disassemble highly entangled states. If the state assembly is non-adiabatic, we imperfectly prepare the target states of our quantum fluid; if the disassembly is non-adiabatic, then we imperfectly map them back to the initial qubits. One might expect that such non-adiabaticity would simply reduce the contrast of the resulting manybody Ramsey fringe, but the reality is more subtle: To the extent that the non-adiabaticity is minimal, only a small amount of population is transferred out of the instantaneous eigenstates during assembly, of which some fraction is transferred back during disassembly (see Fig. 3A). This has the effect of adding new frequency components to the Ramsey fringe, which provide information about the excitation spectrum of the manybody system.

We investigate this phenomenon in Fig. 3 (B to D) by varying the length τ of our adiabatic assembly and disassembly ramps. In Fig. 3B, we plot the Fourier transform of the Ramsey fringe for the slowest (upper) and fastest (lower) ramps: When the ramp is slow compared with manybody gaps (τ = 1 μs ≫*J*^−1^), we observe a single-frequency component in the Ramsey spectrum indicating preparation of a superposition of only a single pair of states. When the ramp is fast (τ = 1 ns ≪ *J*^−1^), we observe numerous frequency components in the Ramsey spectrum indicating that we have prepared numerous pairs of states that then interfere. In Fig. 3C, we plot the Fourier spectrum as we tune the ramp time τ over three decades, observing the appearance of increasing numbers of peaks as the ramp gets faster. Repeating this experiment with more particles (Fig. 3D) reveals fewer total peaks, despite the larger state space accessible with more particles. This occurs because the number of accessible states grows so rapidly that for all but the slowest ramps the features overlap and smear into a continuum.

In practice, achieving the best spectroscopic resolution for the Ramsey signal frequency is a balancing act between (i) particle loss/dephasing if the protocol takes too long compared to the photon *T*_1_/*T*_2_ (see the “Device parameters” section in Materials and Methods) and (ii) reduction in the spectral weight of the correct Fourier peak if the adiabatic ramp time τ is too small and the wrong manybody states are prepared. To circumvent decoherence in larger systems, we choose faster ramps that induce some diabatic excitation without obscuring the correct Fourier feature.

### Extracting thermodynamic observables

Having demonstrated the ability to interferometrically extract the energy difference of arbitrarily chosen manybody states, we now apply the technique to the measurement of thermodynamic observables of a quantum fluid. To do this, we rely upon the fact that thermodynamic quantities like the chemical potential and the pressure may be understood as the rate of change of the system energy with density, and thus particle number, a quantity that our manybody interferometry technique probes directly.

**Fig. 4.**
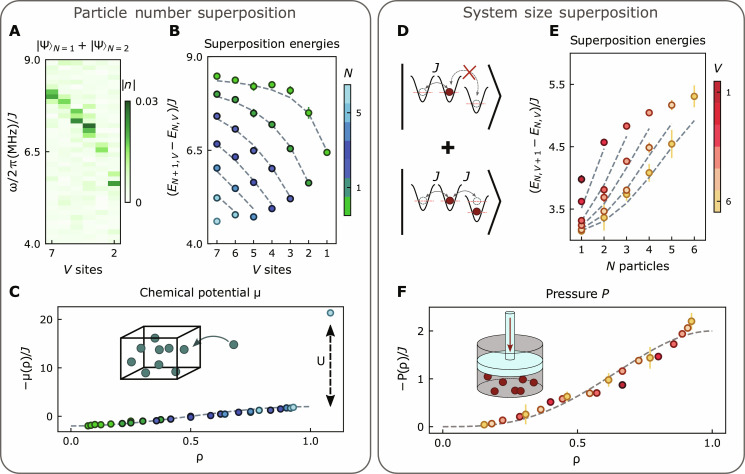
Spectroscopic probes of thermodynamics. Manybody Ramsey interferometry offers new ways to characterize synthetic quantum matter: (**A** to **C**) The chemical potential μ = *E*_*N*+1,*V*_ − *E*_*N*,*V*_ quantifies the energy required to add a particle to a manybody system, and we measure it by interfering states of different particle number. (A) is a sample dataset showing the Ramsey spectrum for the superposition of *N* = 1,2 particles as we vary the total system size *V*. For each *V*, the chemical potential μ is assigned to the frequency of maximal spectral density, which we plot in (B) for all fillings up to unit filling *N* = 0…*V* − 1, and all system sizes *V* = 1...7. Exact values calculated from numerics using our measured device’s parameters are plotted with gray dashed lines. In (C), we replot all data versus the density ρ ≡ *N*/*V*, finding a collapse onto a universal sinusoidal form (gray dashed line) consistent with a free-fermion model (*50*). (**D** to **F**) The pressure *P* = *E*_*N*,*V*+1_ − *E*_*N*,*V*_ quantifies the energy required to change the system size, and we measure it by interfering states of different system size. We achieve controlled superpositions of system sizes using the approach shown in (D): the controlling site is *U*-detuned such that when it is empty, it is energetically inaccessible, reducing the system size by a single site; when it is filled, it becomes accessible and accordingly increases the system size. Using this site as the control in a manybody Ramsey experiment allows us to extract the energy difference between *N* particles melted into *V* + 1 versus *V* sites (see the Supplementary Materials, section B). Performing this protocol for different volumes *V* and particle numbers *N* produces the raw data in (E), which we rescale versus density in (F), again finding agreement with a free-fermion theory. Error bars, where larger than the data point, reflect the SEM (see the Supplementary Materials, section K).

The chemical potential is the energy required to add a particle to the manybody system at fixed system size *V*, $\mu =E_{N+1,V}-E_{N,V}\approx {\left.\frac{\partial E}{\partial N}\right|}^{V}$: We thus measure μ by performing manybody Ramsey interferometry between the *N* and *N* + 1 particle ground states. In Fig. 4A, we demonstrate this measurement for the superposition of *N* = 1 and *N* = 2 particles as we vary the number of accessible sites *V*. At each *V*, the Fourier frequency with the largest oscillation amplitude corresponds to the chemical potential. In Fig. 4B, we plot this chemical potential versus *V*, repeating the measurements for particle numbers from an empty system *N* = 0 to a filled system *N* = *V* − 1. In Fig. 4C, we replot the data versus the density $\rho \equiv \frac{N}{V}$, finding collapse onto a universal (intensive) form μ = −2*J*cos(πρ): Adding particles reduces the volume available to each particle, increasing the uncertainty-induced kinetic energy required to add it to the system. These data are consistent with a free-fermion model (see the Supplementary Materials, section E) (*50*, *51*) modulo small system-size corrections (see the Supplementary Materials, section F.1). Beyond unit filling, we observe an additional energy cost *U* per particle reflecting the incompressibility of the unit-filled Mott-insulating state (*50*).

The pressure is the force required to maintain the fluid at fixed size, or equivalently the energy required to reduce the system size $P=E_{N,V}-E_{N,V+1}\approx -{\left.\frac{\partial E}{\partial V}\right|}^{N}$. To directly measure the pressure, we thus need to perform manybody interferometry between systems of different sizes rather than different particle numbers. We achieve this by engineering our ancilla qubit to control the system size: As shown in Fig. 4D, the ancilla site is detuned in energy by *U*, ensuring that when it is empty particles cannot tunnel onto it (reducing the system size by one site), and when it is occupied particles can tunnel (Bose-enhanced tunneling onto the occupied ancilla is compensated by Floquet engineering; see the Supplementary Materials, section B). The measured pressures for all particle numbers and system sizes are shown in Fig. 4E. They are replotted versus density in Fig. 4, demonstrating that higher densities lead to more uncertainty pressure, again in agreement with a free-fermion model anticipated to describe the 1D hardcore bosons in our experiments (see the Supplementary Materials, section C).

## DISCUSSION

We have introduced a probe of synthetic quantum matter that accesses manybody observables by blurring the boundary between state preparation and measurement. Rather than first preparing a state and then characterizing it, we instead control what manybody state we prepare with an ancilla qubit, coherently reverse the preparation procedure to disentangle the ancilla from the manybody system, and sandwich this process within an ancilla Ramsey sequence. This manybody Ramsey interferometry protocol enables direct measurement of energy differences of different eigenstates of the same system, as well as the same eigenstate of different systems. We use it to directly extract thermodynamic properties of a quantum fluid.

Because the manybody Ramsey protocol relies upon reversible adiabatic assembly of manybody states (*32*), it requires only a small factor more coherence time than adiabatic state preparation. In other words, if you can build a state, you can characterize it with manybody Ramsey interferometry. In long ramps where contrast is strongly suppressed by decoherence, one can trade off a faster ramp for some diabatic excitations, increasing the contrast of the desired Fourier component despite introducing some other low-amplitude Fourier features as discussed in the “Probing the excitation spectrum” section and in the Supplementary Materials, section B.1.

Marrying these techniques with recent advances in topological quantum matter (*52*) will enable probes of fractional statistics (*53*); applying the techniques to glassy (*54*, *55*) or time-crystalline (*56*, *57*) phases has the potential to shed light on their structure. We anticipate opportunities to apply manybody Ramsey interferometry to cold atoms, particularly in topological (*19*) or fermionic sectors (*58*). Marrying this tool with a quantum Fourier transform suggests yet more efficient approaches to quantum sensing in manybody systems (*40*). More broadly, this work invites the question: What observables become accessible when multiple ancillas are entangled with, and then disentangled from, a quantum material? We envision a future where hitherto unimagined observables are probed by entangling quantum matter with small quantum computers.

## MATERIALS AND METHODS

### Device fabrication

The sample is a 10 × 20 mm sapphire chip with a tantalum base layer and aluminum Josephson junctions and SQUID loops. Our substrate is a 450-μm-thick C-plane sapphire wafer that has been annealed at 1500°C for 2 hours, solvent-cleaned, etched in 80°C Nano-Strip for 10 min, and then etched again in 140°C sulfuric acid to fully remove all contaminants. The large-scale features of the device are defined using optical lithography. The base layer is 200 nm of tantalum deposited at 800°C, then patterned with a direct pattern writer (Heidelberg MLA 150), and wet-etched in hydrofluoric acid. Next, the junctions and SQUID loops are defined with electron beam lithography, using a methyl methacrylate - poly methyl methacrylate (MMA-PMMA) bilayer resist, written on a Raith EBPG5000 Plus E-Beam Writer. The Al/AlO*_x_*/Al junctions are e-beam evaporated in an angled evaporator (Plassys MEB550). Before Al deposition, Ar ion milling is used on the exposed Ta to etch away the Ta oxide layer to ensure electrical contact between the Ta and Al layers. The first layer of Al (60 nm, deposited at 0.1 nm/s) is evaporated at an angle of 30° to normal, followed by static oxidation in O_2_ for 24 min at 50 mbar. The second layer of Al (150 nm, 0.1 nm/s) is then evaporated at 30° to normal but orthogonal to the first layer in the substrate plane to form the Manhattan-style junctions.

### Device parameters

The optimal lattice frequency varied on a scale of weeks depending on the frequency distribution of low lifetime defects. For data taken for Fig. 2, we used lattice frequency 5.31 GHz, for Fig. 3 we used lattice frequency 4.820 GHz, and for Fig. 4 we used lattice frequency 5.0 GHz. Our qubit *T*_1_s and *T*_2_s similarly varied over time, with the average *T*_1_ = 40 μs and average *T*_2_^*^ = 1.3(μs). Our qubit *T*_2_ times when all the qubits are on resonance improve, since, because of the avoided crossings, the eigenvalue versus flux curves become flatter (we generate our own sweet spot). We did not quantitatively measure this effect.

### Microwave wiring

The device is mounted and wire-bonded to a multilayer copper printed circuit board. The ground plane around the device features is also heavily wire-bonded to avoid slotline modes and to fully connect the ground plane across the chip. The device is enclosed in an oxygen-free high thermal conductivity (OFHC) copper mount designed to eliminate spurious microwave modes near our operating frequencies.

The packaged sample is mounted to the base plate of a BlueFors dilution refrigerator at a nominal temperature of 8 mK. A solenoid of coiled niobium-titanium (NbTi) wire is fixed to the packaged sample to provide a global bias field with little heating, useful for getting close to desired lattice flux bias point without driving too much current through the DC flux lines. The sample is placed in a heat-sunk can consisting of a thin high-purity copper shim shield, followed by a high-purity superconducting lead shield, followed by two μ-metal shields (innermost to outermost) to provide additional shielding from radiation and external magnetic fields. Control and probe microwave signals run from a room-temperature measurement setup through microwave coaxial cables and DC twisted pair wires into the shielded sample. Superconducting NbTi lines carry signal from the sample back to the room temperature homodyne setup. See the supplement of our previous work (*32*) for further details on cryogenic and room-temperature wiring.

See the supplement of our previous work (*32*) (which used the same device and setup) for further details on cryogenic and room-temperature wiring device parameters, DC and RF (radio frequency) flux calibrations, flux line transfer function correction, and readout methods. The RF cross-talk measured at 100 MHz for experiments involving modulation in this work is lower, close to 2 to 3%.

## Supplementary Materials

### This PDF file includes:

Supplementary Text Figs. S1 to S9 Table S1 References
