# Supplementary material for: Manybody interferometry of quantum fluids
Source: Sci Adv. 2024 Jul 19;10(29):eado1069. doi: 10.1126/sciadv.ado1069 (PMC11259156; doi:10.1126/sciadv.ado1069)
Supplement: Supplementary file 1 — Supplementary Text Figs. S1 to S9 Table S1 References [file sciadv.ado1069_sm.pdf]

Science Advances
AAAS

Supplementary Materials for

Manybody interferometry of quantum fluids

Gabrielle Roberts *et al.*

Corresponding author: David I. Schuster, dschus@stanford.edu

Sci. Adv. **10**, eado1069 (2024)
DOI: 10.1126/sciadv.ado1069

This PDF file includes:

Supplementary Text
Figs. S1 to S9
Table S1
References

Supplementary Information

A Ramsey Interferometry Measurements

A Ramsey interferometry experiment on a single qubit measures that qubit's ω_{01} frequency (relative to the frequency of the tone used to drive the qubit). The experiment sequence is as follows. First, the qubit is driven into an equal superposition of $|0\rangle$ and $|1\rangle$ with a microwave $\pi/2$ pulse. The two states evolve relative to each other for time T with phase $\phi = \omega_{01}T$. A second $\pi/2$ pulse is applied, mapping oscillations around the equator of the Bloch sphere to population oscillations. The qubit population is read out, producing a fringe oscillating at $\omega_{01}T$ minus the frequency of the drive that the qubit is referenced to.

Manybody Ramsey works in a similar fashion. When measuring the energy difference of states with N vs $N-1$ particles when the qubits are all on resonance at the lattice frequency, the energy difference is approximately one lattice photon, 5.2 GHz. However, the qubit drive to which the energy difference is compared is at the frequency of the ancilla in the staggered position, on the order of hundreds of MHz detuned from the lattice frequency. The time resolution of our AWG is 1 ns; the maximum frequency we can physically record without aliasing is 500MHz; the maximum frequency we can comfortably measure is 250 MHz. In practice, we use a sampling rate of $3-4$ μs, which brings our maximum measurable frequency even lower. We thus add a time-dependent virtual phase to our second Ramsey $\pi/2$ pulse in order to virtually change the frequency of the qubit drive used as reference, bringing the measured oscillation frequency below the aliasing limit.

While measuring states with V vs $V-1$ sites, at first glance it seems like the energy differences involved should be within the same particle manifold. However, because of the way we measure volume superpositions (see SI B), the states we compare do differ by a particle, so we measure Ramsey oscillation as above.

To achieve appropriate frequency resolution for experiments involving examining Ramsey Fourier peaks, we record the Ramsey fringe for a minimum of $T = 800$ ns in steps of $3 - 4$ ns for a frequency resolution of at maximum 1.25 MHz (Fig. 3 2.7 µs, Fig. 4 804ns). We choose our time resolution to balance experiment time (long experiments suffer more from frequency drift) and retaining the ability to distinguish frequencies we care about without encountering frequency aliasing.

B Thermodynamic Observable Measurement Details

B.1 Chemical Potential

To extract chemical potential, we compare the energy differences of states with N vs $N - 1$ particles in a given volume V for a range of volumes and particle numbers by extracting the interference fringe between the relevant states. We compare highest energy eigenstates of each particle manifold, which map to the ground states of a repulsive-U model (see SI D). Depending on the number of particles, volume, and adiabatic ramp time, this measurement is operated at the edge of the coherence time of our qubits. To extract consistent signal, we play a few tricks. First, which of the qubits participate in the highest energy eigenstate in the disordered configuration depends on which qubits we place highest in frequency. Therefore, we can arbitrarily choose which qubits to use for any given eigenstate (limitations: neighbors need to start properly detuned so that disordered state is separable). For volumes V less than 7, we can also choose different contiguous sets of qubits as our volume for that experiment. To find the configuration with best coherence and least noise for a given superposition, we cycle through qubits, volume sets (where possible), and ramp times, until we hit a combination that has a peak of prominence 6σ above background noise after applying a digital low-pass filter cutting off frequency components below 20 MHz with the Ramsey virtual frequency chosen to fall between 30 MHz and 100 MHz (see SI A). We then repeat the experiment to acquire error

bars on peaks. In some cases, to get a clear peak despite coherence limitations we had to ramp faster than would be required for full adiabaticity, inducing some extra Fourier components from diabatic excitations. An example of a single peak-finding run is given in Fig. S1. For several of the traces, the ramp time we choose to acquire a high signal-to-noise peak results in smaller additional peaks from diabatic excitations. Notably, in any given data collection run, we were not able to find ramp times to find resolvable peaks for all particle/volume combinations; in Fig. S1, for example, $N = 5$ vs 6 particles in 7 sites shows no signal. Consistently, though, when running two or three such runs spaced a few hours apart, we are able to acquire all peaks. This variability between runs (and qubit configurations) suggests that our coherence in these cases is most limited by moving low-coherence points along the adiabatic frequency trajectory (known as two-level-systems, or TLS).

In order to have small qubit-readout crosstalk, when reading out we place the qubits in a stagger with nearest-neighbor detuning $\gg U$. For Fig. 2 and Fig. 3 superpositions were prepared directly from this point. When preparing superpositions of states for Fig. 4 however, since some states at higher densities involve nearest neighbors, to avoid unwanted crossings with the U-band, qubits were first populated with photons, then rapidly jumped from their readout position to a stagger with nearest-neighbor detuning less than U and qubit position chosen to minimize hybridization. We found we suffered minimally from Landau-Zener transitions (i.e. unwanted population transfer between nearest-neighbors during rapid ramps) and were consistently able to choose staggers where eigenstates still consisted of local qubit states. The adiabatic melt was then performed from this smaller stagger.

Data is plotted with ω_{lat} subtracted off.

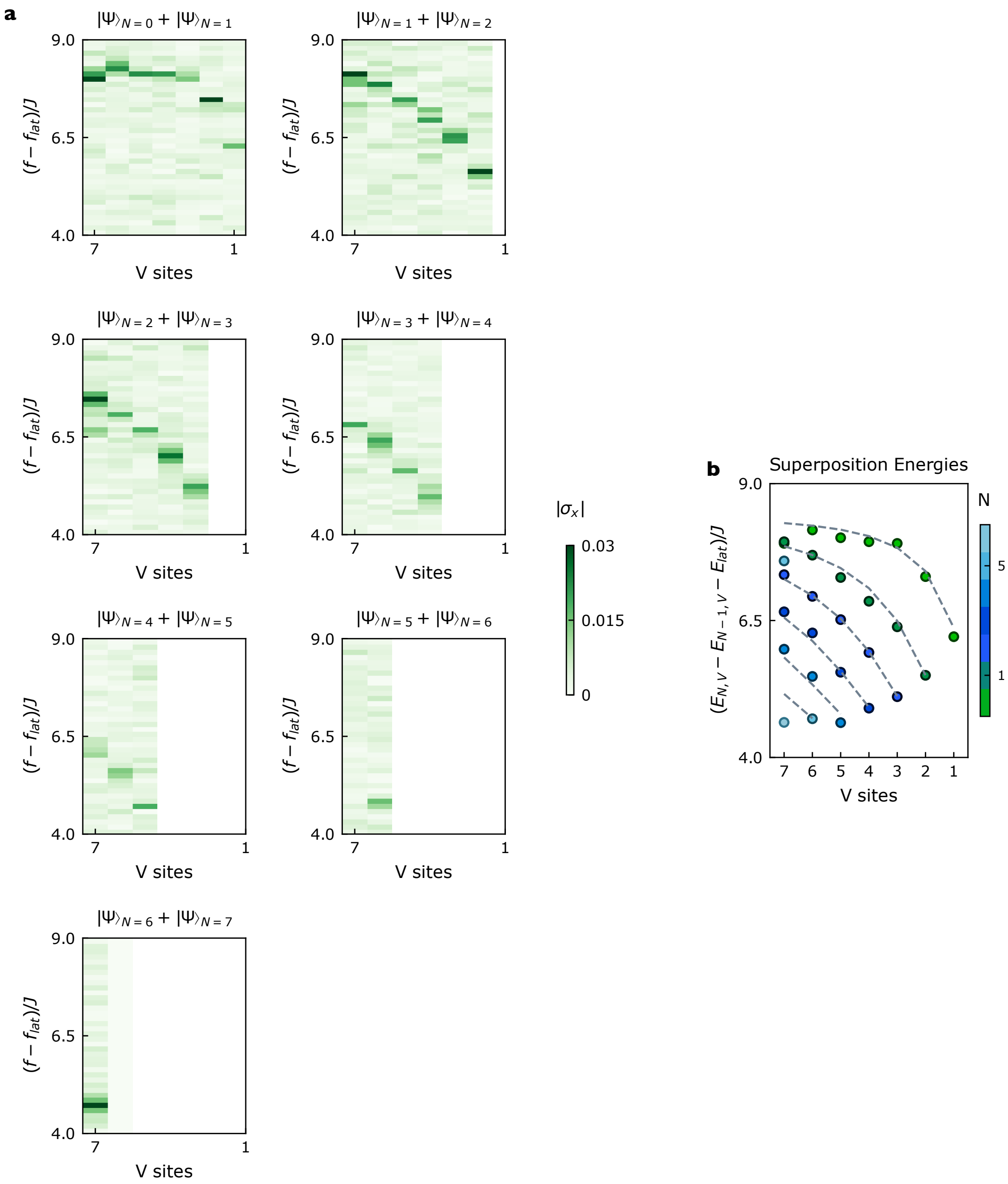

Supplemental Information Fig. S1: **Example of peak extraction run for chemical potential**. In **a**, we plot Fourier signal for each particle number superposition at different volumes. In **b**, we plot the extracted peaks.

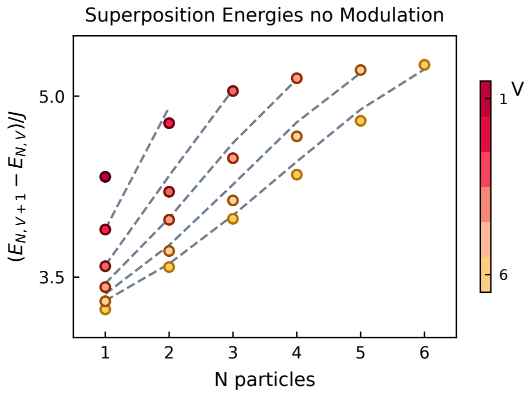

Supplemental Information Fig. S2: **Interferometry of volume superpositions without modulating to correct for Bose-enhancement**. We measure $E_{N,V+1} - E_{N,V}$ for different particle numbers and volumes without modulating the edge qubit to correct for Bose-enhancement. Our measurements line up well with disorder-free numerics (grey dashed lines), confirming that there is low frequency disorder between the qubits before adding the extra complexity of the modulation step. Error bars, where larger than the data-point, reflect the S.E.M.

B.2 Pressure

We apply a similar procedure to measure pressure, with one extra step. As a reminder, to measure the energy of N particles in V vs $V+1$ sites in order to extract pressure, we detune an edge qubit by the anharmonicity, and place that qubit into a superposition of $|0\rangle$ and $|1\rangle$ to make the qubit $|2\rangle$-state in a superposition of being accessible vs not to the rest of the lattice. However, when tunneling into the $|2\rangle$-state, the tunneling is Bose-enhanced by a factor of $\sqrt{2}$. As a sanity check, we measure energy differences in this configuration and compare to an exact numerical model, where we indeed see the spectrum affected by the Bose-enhanced tunneling to the edge site (see Fig. S2). To suppress this extra tunneling, we frequency modulate (*60*) the edge qubit. When frequency modulating, in the rotating frame, the qubit effectively lives with amplitude given by $J_m(\frac{\epsilon}{2\nu_{sb}})$ at the base frequency plus multiples m of the modulation frequency, where J is a Bessel function of the first order, ϵ is the strength of modulation in qubit frequency, and ν_{sb} is the sideband frequency. If one modulates at the right amplitude, one can

engineer a $\sqrt{2}$ suppression of tunneling at the base frequency.

We choose to modulate at 100 MHz in order to have the higher sidebands be far enough detuned from the lattice to not affect its physics. Because the flux vs current curve of the qubits isn't linear, flux modulating also gives a DC offset term given by $\delta_{DC} = \frac{\epsilon_\phi^2}{4}\frac{d^2\nu_{01}}{d\phi^2}$ where ϵ_ϕ is the amplitude of modulation in terms of flux ϕ applied to the qubit and ν_{01} is the frequency of the qubit $|0\rangle$ to $|1\rangle$ transition. We calibrate the flux drive amplitude by fitting single qubit Ramsey frequency components to corresponding Bessel functions and measuring DC offset, see Fig. S4.

Applying the modulation from the start of the ramp of the disorder makes adiabaticity quite hard to achieve; the higher sidebands of the modulated qubit can interact with neighbors during the ramp. We found it easier to instead adiabatically ramp qubits onto resonance, and then adiabatically turn on the modulation. In the code loop aiming to find good parameters, we also varied ramp time for turning on modulation.

Similarly to the data set for chemical potential, to get signal for a given superposition, we cycle through qubits, volume sets (where possible), and ramp times, until we hit a combination that has a peak of prominence 6σ above background noise after applying a digital low-pass filter cutting off frequency components below 20 MHz with a Ramsey virtual frequency chosen to fall between 30 MHz and 100 MHz (see SI A). The exception is for large volume and particle number: for $V = 7$ vs $V = 6$ sites at filling $N = 4, 5, 6$ there are no peaks 6σ above background noise. We instead choose peaks 4σ above the background noise and apply a 30 MHz low pass filter (higher filter since because of our lower peak cutoff we are more susceptible to low frequency noise, and because these energy differences are expected to be higher in frequency).

B.3 Discrepancy in Pressure Data at High Densities

In Fig. 4E. and F., data at high densities overestimate numerics calculated from system parameters (as well as free-fermion theory) by a few MHz. However, data for volume superpositions

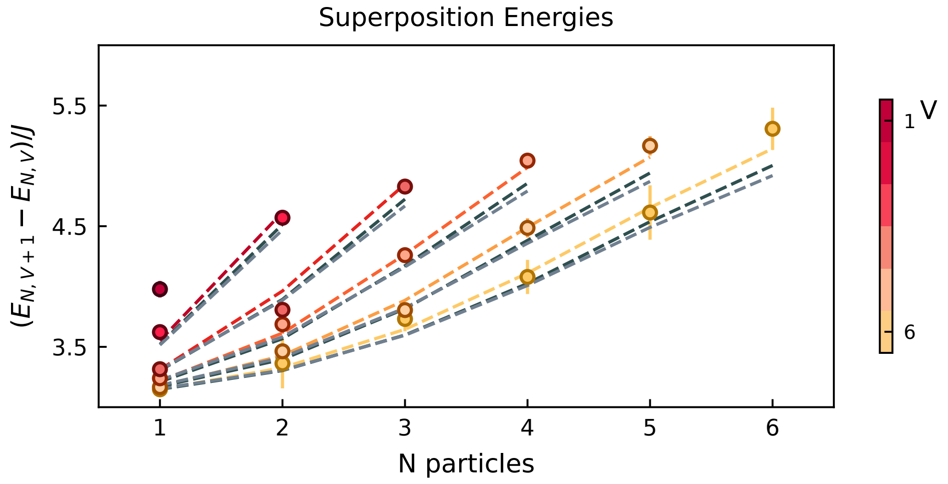

Supplemental Information Fig. S3: **Discrepancy in Pressure Data at High Densities**. Data (circles) overestimate exact numerics calculated for comparing energies of N particles in V vs $V+1$ our system if we were actually able to remove sites (light grey). Computing numerics for our system when detuning and modulating an edge site (dark grey line), the experimental method used for data in Fig. 4E. and F., produces results which are still lower than data. Mis-calibrating the constant frequency offset on the edge site generated by modulation is one possibility for explaining the discrepancy; numerics calculated for a detuned and modulated edge site with a 1.5MHz frequency offset (colored dashed lines) best matches our data.

without modulation to correct for the Bose-enhanced tunneling at the edge site match numerics quite well (see Fig. S2). So, the issue is with the addition of the modulation correction.

Investigating in numerics, one possibility is that we did not correctly account for the constant frequency offset which was generated on the edge site from the modulation. An offset of 1.5 MHz is the best match to our data, plotted in Fig. S3.

Considering other sources of error, it seems unlikely that we modulated at the wrong amplitude (which would lead to the wrong tunneling correction) as doing so leads to a discrepancy at all densities. Modulation crosstalk between sites is minimal (measured at $\approx 4\%$) and only produces shifts of a few 100s of kHz in numerics. Diabatic excitations from ramping on the modulation would both show up as separate peaks, and at highest densities would cause the data to underestimate numerics. (At highest densities, the energies being compared are N particles in $V = N$ vs $V = N + 1$ sites. Since there is only one state in the $V = N$ manifold with significant matrix elements connected to our drives, diabatic excitations would manifest as accidentally transitioning into lower energy states of the $V = N + 1$ manifold, leading to a lower measured energy difference.) Deviations between (a) actually removing sites from the system vs (b) U-detuning an edge site and modulating to correct the Bose-enhanced tunneling are also only on the order of 100s of kHz.

B.4 Derivative

The x-axis point for a measurement comparing $y(x + 1) - y(x)$ is $x + 0.5$. For example, for $E(N + 1, V) - E(N, V)$, the density point in the plot is $\rho = (N + 0.5)/V$.

C TG Gas and Fermioniziation

Girardeau showed in 1960 that there is a one-to-one correspondence between impenetrable (i.e. strongly interacting) bosons confined in 1D and non-interacting spinless fermions (*51*). A

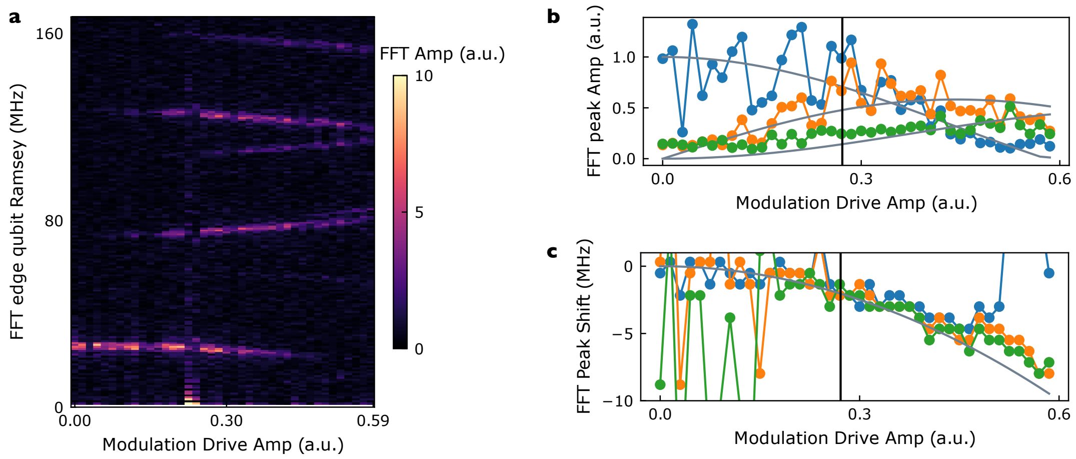

Supplemental Information Fig. S4: **Modulation Calibrations**. We frequency modulate $Q6$, the lattice edge qubit, at 100 MHz and measure the corresponding Ramsey signal at different modulation amplitudes. In **a**, the Fourier transform of the corresponding $Q6$ Ramsey fringes are plotted vs modulation drive amplitude, with a 20 MHz virtual offset. The peaks correspond to the different sidebands, and the peaks' shift in frequency as a function of amplitude is the DC-offset caused by the the non-linearity in the qubit flux-frequency curve. Several of the peaks at higher frequency are frequency folded/aliased because of finite sampling bandwidth. In **b**, the first peaks at the first three sidebands are extracted and their amplitude plotted. By fitting these amplitudes to the expected analytic expressions (Bessel functions), we extract the modulation amplitude where the signal at the base qubit frequency (blue) is suppressed by a factor of $\sqrt{2}$, marked by the black vertical line. Using the same fit parameters as for the Bessel functions, we also fit the DC shift for the range of modulation drives in **c**. This fit is used to extract and correct for the DC shift detuning when modulating by tuning the qubit to compensate.

collection of bosons in the regime where this mapping holds is called a Tonks-Girardeau (TG) gas.

This mapping between strongly interacting bosons and non-interacting fermions is quite useful, as closed-form expressions for thermodynamic quantities for non-interacting fermions are straightforward to analytically derive. The theory curves presented for pressure and chemical potential in Fig.4 come from the analytic expressions for non-interacting fermions-on-a-lattice. The accuracy of the Girardeau's mapping depends on the relative strength of the interaction term and the tunneling term; terms that break the mapping (to first order they appear like fermion interactions) scale as powers of J/U (*61*). In this experiment J/U = 0.04, and all corrections to energy/chemical potential/pressure are small, on average 1.2 MHz, the same order of magnitude as deviation from exact numerics ($700 - 800$ kHz, see SI F.2.

While we use an exact numerical model for the dashed grey theory curves for expected density profiles in Fig.2, we can also use TG gas formalism to solve for these density profiles as well. We assume a 1D ground state many-body wave function of the Bijl-Jastrow (*62*) form $\Psi_B(\mathbf{x}) = \phi(\mathbf{x})\varphi(\mathbf{x})$, for $\mathbf{x} = (x_0, x_1, \ldots, x_6)$, where the open boundary condition of our lattice (i.e. the potential well) is captured by the component $\phi(\mathbf{x}) = \prod_{i=0}^{6} \cos(\pi x_i/L)$, and the two-particle component $\varphi(\mathbf{x}) = \prod_{i<j} |x_i - x_j|$ gives the TG gas impenetrable boson requirement (*50*). Using this trial wavefunction, we calculate density profiles for different particle numbers in the potential, and find very close agreement between exact diagonalization of our lattice and the results from the analytic wavefunction (*32*).

D Ground vs Highest State

We want to explore the properties of the ground states of the repulsive Bose-Hubbard model $\mathbf{H}_{\mathrm{BH}}(t)/\hbar = J\sum_{\langle i,j\rangle} a_i^{\dagger}a_j + \frac{U}{2}\sum_i n_i\,(n_i - 1) + \sum_i (\omega_{lat} + \delta_i(t))n_i$. However, the physical Hamiltonian implemented in our experiment has the sign of U and J flipped (ie, we are realizing

the attractive Bose-Hubbard model); $H_{\text{physical}} = -H_{\text{BH}}$. Because the two Hamiltonians differ only by a minus sign, the eigenstates are the same, just with flipped eigenvalues. Since our system is dissipation-less, the dynamics and observables of the highest excited state of our physical model are the same as those of the ground state of the repulsive model (with reversed time and negative values for thermodynamic/energy observables because of the minus sign). Thus, we measure observables of the highest excited state of our system, which maps onto the ground state of a repulsive Bose-Hubbard Hamiltonian. For the rest of the supplement, we refer to the state we measure as the "ground state"; the analytic values we derive for the ground state thermodynamics quantities match our data up to a minus sign.

E Thermodynamic Analytics of 1D Fluids

In this section, we motivate and derive the analytic thermodynamic expressions plotted in grey in Fig. 4 assuming our particles act like non-interacting fermions on a lattice, as motivated in SI C. Our data matches these expressions up to a minus sign, as we measure the highest excited state of our system.

In the experiment described in this paper, we compare ground state energies of various particle and volume manifolds. This means we measure ground state observables, i.e. chemical potential, pressure, etc. for $T = 0$ and fixed entropy. The following thermodynamic calculations can be done assuming constant temperature and entropy.

The energy eigenvalues of a 1D fermionic lattice with V sites, tunneling $+J$, and open boundary conditions is

$$E_k = 2J\cos\left(\frac{\pi k}{V+1}\right) \tag{S2}$$

where k are quasi-momenta. The ground state energy of N fermions in a lattice, by the Pauli exclusion principle, is the sum of all single-particle energies from the lowest energy state up,

here from $k = N$ to $k = N - V$:

$$E_N = \sum_{k=N}^{N-V} 2J\cos\left(\frac{\pi k}{V+1}\right) = -J\left[\csc\left(\frac{\pi/2}{V+1}\right)\sin\left(\frac{\pi(N+\frac{1}{2})}{V+1}\right) - \frac{1}{2}\right]. \tag{S3}$$

This expression is used to derive thermodynamic quantities below.

To calculate E in the thermodynamic limit, we take ρ constant and send N and V to infinity:

$$\lim_{N,V\to\infty} E_N = \lim_{N,V\to\infty} -J\left[\csc\left(\frac{\pi/2}{V+1}\right)\sin\left(\frac{\pi(\rho V+\frac{1}{2})}{V+1}\right) - \frac{1}{2}\right] = \frac{-2JV\sin(\pi\rho)}{\pi}. \tag{S4}$$

The chemical potential at constant entropy is defined as $\mu = \frac{\partial E}{\partial N}\Big|_{S,V}$. Plugging in the expression for E from S3 gives:

$$\mu = \frac{-J\pi}{V+1}\csc\left(\frac{\pi/2}{V+1}\right)\cos\left(\frac{\pi(N+\frac{1}{2})}{V+1}\right) \tag{S5}$$

.

In the thermodynamic limit, sending N and V to infinity while holding $\rho \equiv \frac{N}{V}$ constant,

$$\lim_{N,V\to\infty} \mu = -2J\cos(\rho\pi). \tag{S6}$$

The pressure at constant entropy is defined as $P = -\frac{\partial E}{\partial V}\Big|_{S,N}$. Plugging in the expression for E from S3 yields:

$$P = \frac{J\frac{\pi}{2}}{(V+1)^2}\csc\left(\frac{\frac{\pi}{2}}{V+1}\right)\left[\cot\left(\frac{\pi/2}{V+1}\right)\sin\left(\frac{\pi(N+\frac{1}{2})}{V+1}\right) - (2N+1)\cos\left(\frac{\pi(N+\frac{1}{2})}{V+1}\right)\right] \tag{S7}$$

.

In the thermodynamic limit, holding ρ constant while sending N and V to infinity,

$$\lim_{N,V\to\infty} P = 2J\left(\frac{\sin(\pi\rho)}{\pi} - \rho\cos(\rho\pi)\right). \tag{S8}$$

Qubit	1	2	3	4	5	6	7
$U_{\text{lattice}}/2\pi$ (MHz)	-236	-235	-209	-234	-236	-231	-225
$(U_{\text{lattice}} - U_{Q1})/2\pi$ (MHz)	-1	0	27	1	-1	4	10

Table S1: **Disorder in Qubit Anharmonicity**

F Souces of Deviation from Non-Interacting Fermion Analytics

F.1 Finite N and V effects

The gray theory curves plotted in Fig. 4 are theory for thermodynamic limit. However, our system is finite in size, and so deviates from this limit. Our data is better captured by finite-size analytics, see Fig S5.

F.2 Finite U/J Effects in a Finite Size System

Zooming in further reveals that at the next level of correction, our data also deviate from even the finite-size correction because of the interaction energy U being finite; the mapping to non-interacting fermions is not perfect, see Fig. S6.

G Anharmonicity Disorder

We measured the Mott insulator gap at all lattice volumes, with $Q1$ as the qubit in a superposition of $|1\rangle$ and $|2\rangle$. However, because of strong variation in the qubits' anharmonicities, lattice configurations beyond $V = 2$ that include $Q2$ were effectively restricted to $Q0$ and $Q1$: $Q2$'s $|2\rangle$ is 26 MHz detuned from its neighbors, causing data at higher particle number to deviate from values expected in a uniform-U case.

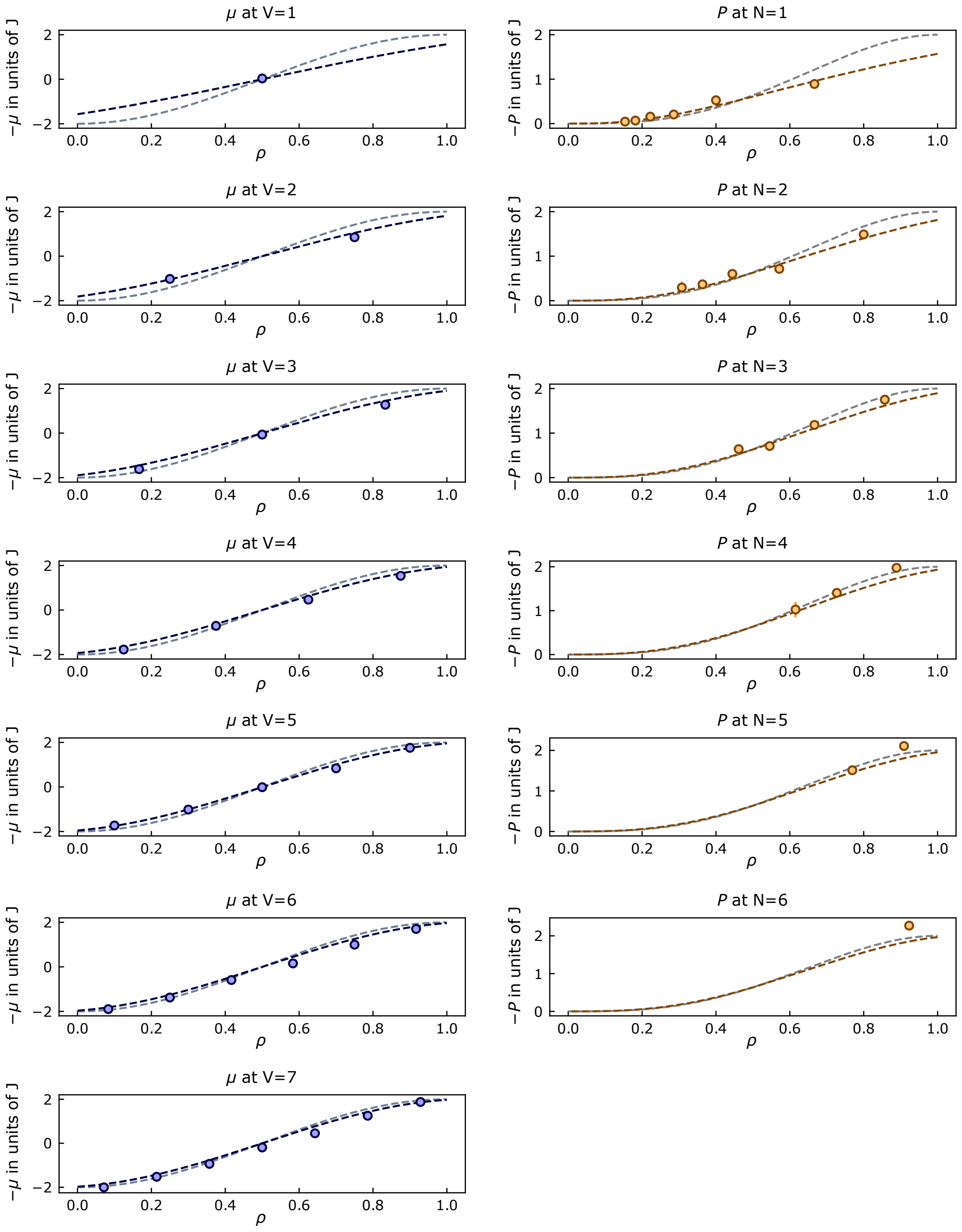

Supplemental Information Fig. S5: **Finite Size N and V Effects in Chemical Potential and Pressure**. Grey dashed lines correspond to free fermion analytics in the thermodynamic limit, dashed colored lines correspond to free fermion analytics in finite size limit indicated by subplot title, and circles correspond to data. Error bars, where larger than the data-point, reflect the S.E.M.

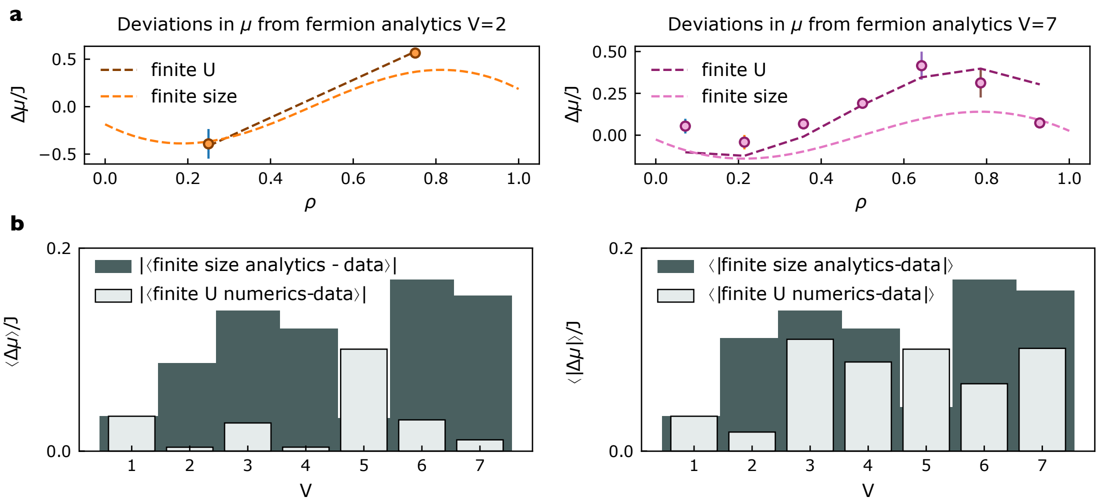

Supplemental Information Fig. S6: **Example of finite U/J effects in chemical potential**. In **a**, we plot the deviation from the infinite size fermion chemical potential for finite size analytics (dashed light line), finite size & finite U numerics (dashed dark line), and our data (circles). In both representative volumes $V = 2$ and $V = 7$, it emerges that while there is scatter in the data, data agrees more closely with finite U & finite size numerics than just the finite size theory. In **b**, in the first panel, for each volume, we measure the average deviation between data and finite size fermion (ie infinite U) analytics (dark grey) and between data and numerics calculated with our finite experimental value of U (light grey). In the second panel, we plot the average of the absolute value of the deviation. These panels illustrate that while the magnitude of the deviation in the data between finite U numerics and infinite U analytics is similar, the data still agrees more closely with finite U numerics. Error bars, where larger than the data-point, reflect the S.E.M.

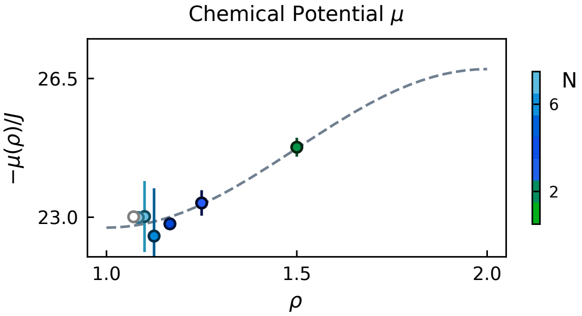

Supplemental Information Fig. S7: **Chemical Potential Above Mott Insulator Gap**. We plot the energy difference between the lattice being fully filled and the lattice being fully filled with one particle band of second excited states U below the first band. Because of Bose enhancement, the effective tunneling in this second band is $2J$, reflected in the grey dashed lines from non-interacting fermion analytics. Because of lattice site disorder, data deviates from free fermion analytics at higher volumes (i.e. lower densities in plot above since particle number here is fixed at $N = 1$). Error bars, where larger than the data-point, reflect the S.E.M.

H Same Particle Manifold Superpositions

We perform a two-qubit gate, preparing the state $\frac{1}{\sqrt{2}}(|01\rangle + |10\rangle)$ between two qubits, to create a superposition of these eigenstates at when the qubits are far detuned from each other, in the stagger configuration. There are several ways to enact a two qubit gate: the way we choose here is to π-pulse one qubit, bring it in resonance with its neighbor for half the J tunneling time, and then jump both qubits back to the stagger. We then proceed with the reversible adiabatic ramp protocol as normal. In Fig. S8**a** we prepare and measure density profiles for two different single particle states at the stagger and at the lattice degeneracy point where all the qubits are on resonance, as well as the density profile for their superposition. We extract the Ramsey trace by measuring $\langle\sigma_{XQ4}\sigma_{XQ5}\rangle$ in Fig. S8**b**, which is within 3 MHz of the expected value.

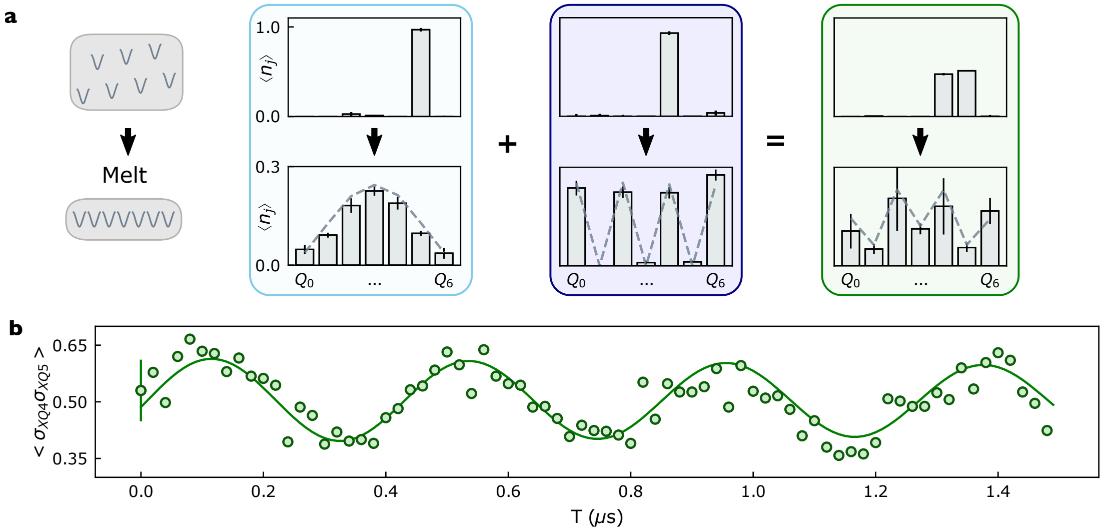

Supplemental Information Fig. S8: **Same Particle Manifold Superpositions**. In **a**, we create and measure density profiles for two different one-particle states, and their superposition, at the disordered stagger position and at the lattice position. We then record the Ramsey beating between the two states by measuring $\langle \sigma_{XQ4}\sigma_{XQ5} \rangle$ in **b**. Representative error bars reflect the S.E.M.

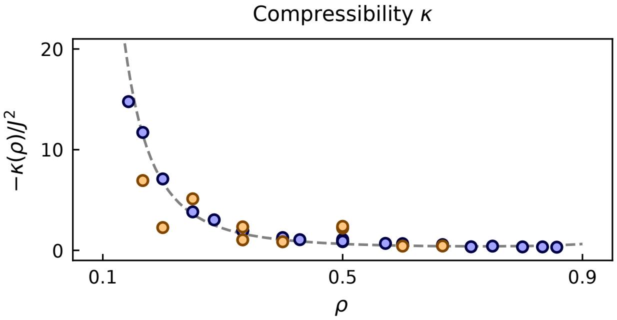

Supplemental Information Fig. S9: **Compressibility**. We plot the quantum fluid compressibility at different density points by differentiating pressure data (orange) and chemical potential data (blue). Error bars, where larger than the data-point, reflect the S.E.M.

I Compressibility

The compressibility reflects how much the pressure changes with volume. In Fig. S9 we compare compressibility computed by differentiating pressure data ($\kappa_s^{-1} = -V\frac{\partial P}{\partial V}$) to compressibility computed by differentiating chemical potential data ($\kappa_s^{-1} = V\rho^2\frac{\partial \mu}{\partial N}|_S$). The agreement that we find further validates that the equation of state is intensive (dependent on particle number and system size only through density).

We calculate the compressibility by taking numeric N and V derivatives of μ and P. The thermodynamic compressibility at constant entropy and particle number is defined as

$$\kappa_s^{-1} = -V\frac{\partial P}{\partial V}\bigg|_{S,N}. \tag{S9}$$

However, with this expression, we can only take a numeric derivative of our pressure data set with respect to volume; it would be be nice to use our chemical potential data set as well.

In the thermodynamic limit, the Gibbs-Duhem expression holds, which states

$$0 = -V\partial P + S\partial T + N\partial\mu. \tag{S10}$$

Since we are at $T = 0$, this implies $\partial P = N/V\partial\mu = \rho\partial\mu$. Therefore,

$$\kappa_s^{-1} = -V\frac{\partial P}{\partial V}\bigg|_{S,N} = -N\frac{\partial \mu}{\partial V}\bigg|_{S,N} = -N\frac{\partial}{\partial V}\bigg|_{S,N}\frac{\partial E}{\partial N}\bigg|_{S,V}. \tag{S11}$$

We flip the order of the derivatives, and once again replace $\partial P = N/V\partial\mu = \rho\partial\mu$ to get the expression $\kappa_s^{-1} = -V\rho^2\frac{\partial^2 E}{\partial N^2}\Big|_{S,V}$ (*63*).

J Disorder Correction

Ensuring that the qubits in the on-resonance lattice configuration are degenerate in frequency is extremely important when measuring pressure and chemical potential. For several densities, the energy differences between ground states are on the order of a few MHz; lattice disorder

can cause significant error in the quantities we are attempting to measure. Using our RF flux crosstalk matrix correction and measured qubit $\omega_{01}(\phi)$ relations, we are able to place qubits within $1-2$ MHz of the desired lattice frequency. To ensure we hit lattice degeneracy to the required precision, we feed back on the local $\omega_{01}(\phi)$ relations by comparing our manybody profiles to expected theory. To ensure our corrections are robust, we feed back on a full set of manybody profiles at $V = 7$: the ground state for $N = 1$, $N = 2$, and $N = 3$. Using this method, we are able to achieve error on the order of $200-400$ kHz, which is the same order of magnitude as experiment-to-experiment qubit frequency drift.

It is also important that the qubits not only be on resonance with each other, but also that we know what lattice frequency they are being placed at after the round of corrections described above. Energy differences between eigenstates of different particle number depend on what energy the particles are at. To ensure we are normalizing correctly (see SI E), it is important that we actually be placing our lattice at the expected frequency. Feeding back on profiles helps correct for relative detuning between qubits, but does not give us insight into the absolute lattice frequency. To measure this quantity, we first measure via standard Ramsey interferometry the frequency of individual qubits brought to the lattice one at a time using the disorder corrections calculated from feedback back on profiles. Usually there is some scatter (from an imperfect crosstalk matrix); we take the average of this scatter. Recent results have shown that using machine learning on flux crosstalk matrices allows for very low disorder (*64*), this would be a better solution going forward.

K Error and Uncertainty Calculations

For each experiment in Fig. 2, we measure 2000 shots, bin the shots, apply relevant confusion matrices, and extract the averaged quantity of interest (in this case, Ramsey traces). We then repeat the experiment 10-11 times. Because of the long ramp times and wait times in the experi-

ment, very small frequency variations experiment-to-experiment cause large phase variations in our measurement. We calculate the starting phase in each trace from fits, and numerically zero the phase. We then calculate the mean of the averages and standard deviation of the resulting traces (i.e. calculate the standard error of the mean, or S.E.M.) for the values and error bars that we report in this figure. The experiment repetitions are performed close in time (typically within a 10-30 minute span) so that our error bars are not affected by slow experimental drifts over hours or days.

For error bars in Fig. 4**B** and **E** come from repeating the procedure described in SI B 3 times and 10-15 times respectively, then taking the mean and standard deviation of the collections of measured peaks. We then propagate the error to gain the error bars in Fig. 2**C**, **F**, and **g**. The exception is the U-detuned point in **C**, where we did not have enough repetitions of the data point. There, the error bar is the variance in a Lorentzian fit to the measured Ramsey peak.

Note that our error bars in Fig.4**B** and **E** are smaller than points' deviation from exact numerical models of our system (average deviation of 700 kHz for particle number superpositions and 800 kHz for volume superpositions). This is likely because of residual frequency disorder (see SI J).

REFERENCES AND NOTES

1. I. Carusotto, A. A. Houck, A. J. Kollár, P. Roushan, D. I. Schuster, J. Simon, Photonic materials in circuit quantum electrodynamics. *Nat. Phys.* **16**, 268–279 (2020).

2. R. Blatt, C. F. Roos, Quantum simulations with trapped ions. *Nat. Phys.* **8**, 277–284 (2012).

3. I. Bloch, J. Dalibard, S. Nascimbene, Quantum simulations with ultracold quantum gases. *Nat. Phys.* **8**, 267–276 (2012).

4. L. W. Clark, N. Schine, C. Baum, N. Jia, J. Simon, Observation of laughlin states made of light. *Nature* **582**, 41–45 (2020).

5. W. S. Bakr, J. I. Gillen, A. Peng, S. Fölling, M. Greiner, A quantum gas microscope for detecting single atoms in a hubbard-regime optical lattice. *Nature* **462**, 74–77 (2009).

6. L. W. Cheuk, M. A. Nichols, M. Okan, T. Gersdorf, V. V. Ramasesh, W. S. Bakr, T. Lompe, M. W. Zwierlein, Quantum-gas microscope for fermionic atoms. *Phys. Rev. Lett.* **114**, 193001 (2015).

7. A. Browaeys, T. Lahaye, Many-body physics with individually controlled rydberg atoms. *Nat. Phys.* **16**, 132–142 (2020).

8. A. H. Karamlou, J. Braumüller, Y. Yanay, A. D. Paolo, P. M. Harrington, B. Kannan, D. Kim, M. Kjaergaard, A. Melville, S. Muschinske, B. M. Niedzielski, A. Vepsäläinen, R. Winik, J. L. Yoder, M. Schwartz, C. Tahan, T. P. Orlando, S. Gustavsson, W. D. Oliver, Quantum transport and localization in 1d and 2d tight-binding lattices. *Npj Quantum Inf.* **8**, 35 (2022).

9. K. Wintersperger, C. Braun, F. N. Ünal, A. Eckardt, M. D. Liberto, N. Goldman, I. Bloch, M. Aidelsburger, Realization of an anomalous floquet topological system with ultracold atoms. *Nat. Phys.* **16**, 1058–1063 (2020).

10. L. Barbiero, C. Schweizer, M. Aidelsburger, E. Demler, N. Goldman, F. Grusdt, Coupling ultracold matter to dynamical gauge fields in optical lattices: From flux attachment to $\mathbb{Z}_2$ lattice gauge theories. *Sci. Adv.* **5,** eaav7444 (2019).

11. A. J. Kollár, M. Fitzpatrick, A. A. Houck, Hyperbolic lattices in circuit quantum electrodynamics. *Nature* **571**, 45–50 (2019).

12. M. Greiner, O. Mandel, T. Esslinger, T. W. Hänsch, I. Bloch, Quantum phase transition from a superfluid to a mott insulator in a gas of ultracold atoms. *Nature* **415**, 39–44 (2002).

13. D. J. Wineland, W. M. Itano, Laser cooling of atoms. *Phys. Rev. A* **20**, 1521–1540 (1979).

14. W. Ketterle, N. Van Druten, Evaporative cooling of trapped atoms, in *Advances in Atomic, Molecular, and Optical Physics* (Elsevier, 1996), vol. 37, pp. 181–236.

15. J. Poyatos, J. I. Cirac, P. Zoller, Quantum reservoir engineering with laser cooled trapped ions. *Phys. Rev. Lett.* **77**, 4728–4731 (1996).

16. J. T. Barreiro, M. Müller, P. Schindler, D. Nigg, T. Monz, M. Chwalla, M. Hennrich, C. F. Roos, P. Zoller, R. Blatt, An open-system quantum simulator with trapped ions. *Nature* **470**, 486–491 (2011).

17. R. Ma, B. Saxberg, C. Owens, N. Leung, Y. Lu, J. Simon, D. I. Schuster, A dissipatively stabilized Mott insulator of photons. *Nature* **566**, 51–57 (2019).

18. J. Simon, W. S. Bakr, R. Ma, M. E. Tai, P. M. Preiss, M. Greiner, Quantum simulation of antiferromagnetic spin chains in an optical lattice. *Nature* **472**, 307–312 (2011).

19. J. Léonard, S. Kim, J. Kwan, P. Segura, F. Grusdt, C. Repellin, N. Goldman, M. Greiner, Realization of a fractional quantum hall state with ultracold atoms. *Nature* **619**, 495–499 (2023).

20. M. Hafezi, P. Adhikari, J. M. Taylor, Chemical potential for light by parametric coupling. *Phys. Rev. B* **92**, 174305 (2015).

21. P. Kurilovich, V. D. Kurilovich, J. Lebreuilly, S. M. Girvin, Stabilizing the laughlin state of light: Dynamics of hole fractionalization. *SciPost Phys.* **13**, 107 (2022).

22. Y.-C. He, F. Grusdt, A. Kaufman, M. Greiner, A. Vishwanath, Realizing and adiabatically preparing bosonic integer and fractional quantum hall states in optical lattices. *Phys. Rev. B* **96**, 201103 (2017).

23. C. F. Roos, M. Riebe, H. Haffner, W. Hansel, J. Benhelm, G. P. Lancaster, C. Becher, F. Schmidt-Kaler, R. Blatt, Control and measurement of three-qubit entangled states. *Science* **304**, 1478–1480 (2004).

24. W. Ketterle, D. S. Durfee, D. Stamper-Kurn, Making, probing and understanding Bose-Einstein condensates. arXiv:cond-mat/9904034 (1999).

25. M. Greiner, C. Regal, J. Stewart, D. Jin, Probing pair-correlated fermionic atoms through correlations in atom shot noise. *Phys. Rev. Lett.* **94**, 110401 (2005).

26. T. Yefsah, R. Desbuquois, L. Chomaz, K. J. Günter, J. Dalibard, Exploring the thermodynamics of a two-dimensional Bose gas. *Phys. Rev. Lett.* **107**, 130401 (2011).

27. P. T. Ernst, S. Götze, J. S. Krauser, K. Pyka, D.-S. Lühmann, D. Pfannkuche, K. Sengstock, Probing superfluids in optical lattices by momentum-resolved bragg spectroscopy. *Nat. Phys.* **6**, 56–61 (2010).

28. T. A. Hilker, G. Salomon, F. Grusdt, A. Omran, M. Boll, E. Demler, I. Bloch, C. Gross, Revealing hidden antiferromagnetic correlations in doped hubbard chains via string correlators. *Science* **357**, 484–487 (2017).

29. O. Shtanko, D. S. Wang, H. Zhang, N. Harle, A. Seif, R. Movassagh, Z. Minev, Uncovering local integrability in quantum many-body dynamics. arXiv:2307.07552 [quant-ph] (2023).

30. X. Zhang, E. Kim, D. K. Mark, S. Choi, O. Painter, A superconducting quantum simulator based on a photonic-bandgap metamaterial. *Science* **379**, 278–283 (2023).

31. T. Monz, P. Schindler, J. T. Barreiro, M. Chwalla, D. Nigg, W. A. Coish, M. Harlander, W. Hänsel, M. Hennrich, R. Blatt, 14-Qubit entanglement: Creation and coherence. *Phys. Rev. Lett.* **106**, 130506 (2011).

32. B. Saxberg, A. Vrajitoarea, G. Roberts, M. Panetta, J. Simon, D. I. Schuster, Disorder-assisted assembly of strongly correlated fluids of light. *Nature* **612**, 435–441 (2022).

33. H.-Y. Huang, R. Kueng, J. Preskill, Predicting many properties of a quantum system from very few measurements. *Nat. Phys.* **16**, 1050–1057 (2020).

34. B. Swingle, G. Bentsen, M. Schleier-Smith, P. Hayden, Measuring the scrambling of quantum information. *Phys. Rev. A* **94**, 040302 (2016).

35. K. A. Landsman, C. Figgatt, T. Schuster, N. M. Linke, B. Yoshida, N. Y. Yao, C. Monroe, Verified quantum information scrambling. *Nature* **567**, 61–65 (2019).

36. X. Mi, P. Roushan, C. Quintana, S. Mandrà, J. Marshall, C. Neill, F. Arute, K. Arya, J. Atalaya, R. Babbush, J. C. Bardin, R. Barends, J. Basso, A. Bengtsson, S. Boixo, A. Bourassa, M. Broughton, B. B. Buckley, D. A. Buell, B. Burkett, N. Bushnell, Z. Chen, B. Chiaro, R. Collins, W. Courtney, S. Demura, A. R. Derk, A. Dunsworth, D. Eppens, C. Erickson, E. Farhi, A. G. Fowler, B. Foxen, C. Gidney, M. Giustina, J. A. Gross, M. P. Harrigan, S. D. Harrington, J. Hilton, A. Ho, S. Hong, T. Huang, W. J. Huggins, L. B. Ioffe, S. V. Isakov, E. Jeffrey, Z. Jiang, C. Jones, D. Kafri, J. Kelly, S. Kim, A. Kitaev, P. V. Klimov, A. N. Korotkov, F. Kostritsa, D. Landhuis, P. Laptev, E. Lucero, O. Martin, J. R. McClean, T. McCourt, M. McEwen, A. Megrant, K. C. Miao, M. Mohseni, S. Montazeri, W. Mruczkiewicz, J. Mutus, O. Naaman, M. Neeley, M. Newman, M. Y. Niu, T. E. O'Brien, A. Opremcak, E. Ostby, B. Pato, A. Petukhov, N. Redd, N. C. Rubin, D. Sank, K. J. Satzinger, V. Shvarts, D. Strain, M. Szalay, M. D. Trevithick, B. Villalonga, T. White, Z. J. Yao, P. Yeh, A. Zalcman, H. Neven, I. Aleiner, K. Kechedzhi, V. Smelyanskiy, Y. Chen, Information scrambling in quantum circuits. *Science* **374**, 1479–1483 (2021).

37. R. Fan, P. Zhang, H. Shen, H. Zhai, Out-of-time-order correlation for many-body localization. *Sci. Bull.* **62**, 707–711 (2017).

38. J. Braumüller, A. H. Karamlou, Y. Yanay, B. Kannan, D. Kim, M. Kjaergaard, A. Melville, B. M. Niedzielski, Y. Sung, A. Vepsäläinen, R. Winik, J. L. Yoder, T. P. Orlando, S. Gustavsson, C. Tahan, W. D. Oliver, Probing quantum information propagation with out-of-time-ordered correlators. *Nat. Phys.* **18**, 172–178 (2021).

39. K. Xu, Z.-H. Sun, W. Liu, Y.-R. Zhang, H. Li, H. Dong, W. Ren, P. Zhang, F. Nori, D. Zheng, H. Fan, H. Wang, Probing dynamical phase transitions with a superconducting quantum simulator. *Sci. Adv.* **6,** eaba4935 (2020).

40. V. Vorobyov, S. Zaiser, N. Abt, J. Meinel, D. Dasari, P. Neumann, J. Wrachtrup, Quantum fourier transform for nanoscale quantum sensing *Npj Quantum Inf.* **7**, 124 (2021).

41. K. J. Satzinger, Y.J. Liu, A. Smith, C. Knapp, M. Newman, C. Jones, Z. Chen, C. Quintana, X. Mi, A. Dunsworth, C. Gidney, I. Aleiner, F. Arute, K. Arya, J. Atalaya, R. Babbush, J. C. Bardin, R. Barends, J. Basso, A. Bengtsson, A. Bilmes, M. Broughton, B. B. Buckley, D. A. Buell, B. Burkett, N. Bushnell, B. Chiaro, R. Collins, W. Courtney, S. Demura, A. R. Derk, D. Eppens, C. Erickson, L. Faoro, E. Farhi, A. G. Fowler, B. Foxen, M. Giustina, A. Greene, J. A. Gross, M. P. Harrigan, S. D. Harrington, J. Hilton, S. Hong, T. Huang, W. J. Huggins, L. B. Ioffe, S. V. Isakov, E. Jeffrey, Z. Jiang, D. Kafri, K. Kechedzhi, T. Khattar, S. Kim, P. V. Klimov, A. N. Korotkov, F. Kostritsa, D. Landhuis, P. Laptev, A. Locharla, E. Lucero, O. Martin, J. R. McClean, M. McEwen, K. C. Miao, M. Mohseni, S. Montazeri, W. Mruczkiewicz, J. Mutus, O. Naaman, M. Neeley, C. Neill, M. Y. Niu, T. E. O'Brien, A. Opremcak, B. Pató, A. Petukhov, N. C. Rubin, D. Sank, V. Shvarts, D. Strain, M. Szalay, B. Villalonga, T. C. White, Z. Yao, P. Yeh, J. Yoo, A. Zalcman, H. Neven, S. Boixo, A. Megrant, Y. Chen, J. Kelly, V. Smelyanskiy, A. Kitaev, M. Knap, F. Pollmann, P. Roushan , Realizing topologically ordered states on a quantum processor. *Science* **374**, 1237–1241 (2021).

42. A. Morvan, T. I. Andersen, X. Mi, C. Neill, A. Petukhov, K. Kechedzhi, D. A. Abanin, A. Michailidis, R. Acharya, F. Arute, K. Arya, A. Asfaw, J. Atalaya, J. C. Bardin, J. Basso, A. Bengtsson, G. Bortoli, A. Bourassa, J. Bovaird, L. Brill, M. Broughton, B. B. Buckley, D. A. Buell, T. Burger, B. Burkett, N. Bushnell, Z. Chen, B. Chiaro, R. Collins, P. Conner, W. Courtney, A. L. Crook, B. Curtin, D. M. Debroy, A. del Toro Barba, S. Demura, A. Dunsworth, D. Eppens, C. Erickson, L. Faoro, E. Farhi, R. Fatemi, L. Flores Burgos, E. Forati, A. G. Fowler, B. Foxen, W. Giang, C. Gidney, D. Gilboa, M. Giustina, A. Grajales Dau, J. A. Gross, S. Habegger, M. C. Hamilton, M. P. Harrigan, S. D. Harrington, M. Hoffmann, S. Hong, T. Huang, A. Huff, W. J. Huggins, S. V. Isakov, J. Iveland, E. Jeffrey, Z. Jiang, C. Jones, P. Juhas, D. Kafri, T. Khattar, M. Khezri, M. Kieferová, S. Kim, A. Y. Kitaev, P. V. Klimov, A. R. Klots, A. N. Korotkov, F. Kostritsa, J. M. Kreikebaum, D. Landhuis, P. Laptev, K.M. Lau, L. Laws, J. Lee, K. W. Lee, B. J. Lester, A. T. Lill, W. Liu, A. Locharla, F. Malone, O. Martin, J. R.

McClean, M. McEwen, B. Meurer Costa, K. C. Miao, M. Mohseni, S. Montazeri, E. Mount, W. Mruczkiewicz, O. Naaman, M. Neeley, A. Nersisyan, M. Newman, A. Nguyen, M. Nguyen, M. Y. Niu, T. E. O'Brien, R. Olenewa, A. Opremcak, R. Potter, C. Quintana, N. C. Rubin, N. Saei, D. Sank, K. Sankaragomathi, K. J. Satzinger, H. F. Schurkus, C. Schuster, M. J. Shearn, A. Shorter, V. Shvarts, J. Skruzny, W. C. Smith, D. Strain, G. Sterling, Y. Su, M. Szalay, A. Torres, G. Vidal, B. Villalonga, C. Vollgraff-Heidweiller, T. White, C. Xing, Z. Yao, P. Yeh, J. Yoo, A. Zalcman, Y. Zhang, N. Zhu, H. Neven, D. Bacon, J. Hilton, E. Lucero, R. Babbush, S. Boixo, A. Megrant, J. Kelly, Y. Chen, V. Smelyanskiy, I. Aleiner, L. B. Ioffe, P. Roushan, Formation of robust bound states of interacting microwave photons. *Nature* **612**, 240–245 (2022).

43. P. Roushan, C. Neill, J. Tangpanitanon, V. M. Bastidas, A. Megrant, R. Barends, Y. Chen, Z. Chen, B. Chiaro, A. Dunsworth, A. Fowler, B. Foxen, M. Giustina, E. Jeffrey, J. Kelly, E. Lucero, J. Mutus, M. Neeley, C. Quintana, D. Sank, A. Vainsencher, J. Wenner, T. White, H. Neven, D. G. Angelakis, J. Martinis, Spectroscopic signatures of localization with interacting photons in superconducting qubits. *Science* **358**, 1175–1179 (2017).

44. M. Knap, A. Kantian, T. Giamarchi, I. Bloch, M. D. Lukin, E. Demler, Probing real-space and time-resolved correlation functions with many-body ramsey interferometry. *Phys. Rev. Lett.* **111**, 147205 (2013).

45. J. Zeiher, R. Van Bijnen, P. Schauß, S. Hild, J.-y. Choi, T. Pohl, I. Bloch, C. Gross, Many-body interferometry of a rydberg-dressed spin lattice. *Nat. Phys.* **12**, 1095–1099 (2016).

46. A. A. Houck, H. E. Türeci, J. Koch, On-chip quantum simulation with superconducting circuits. *Nat. Phys.* **8**, 292–299 (2012).

47. N. F. Ramsey, Experiments with separated oscillatory fields and hydrogen masers. *Rev. Mod. Phys.* **62**, 541–552 (1990).

48. T. Kinoshita, T. Wenger, D. S. Weiss, Observation of a One-Dimensional Tonks-Girardeau Gas. *Science* **305**, 1125–1128 (2004).

49. B. Paredes, A. Widera, V. Murg, O. Mandel, S. Fölling, I. Cirac, G. V. Shlyapnikov, T. W. Hänsch, I. Bloch, Tonks-Girardeau gas of ultracold atoms in an optical lattice. *Nature* **429**, 277–281 (2004).

50. M. A. Cazalilla, R. Citro, T. Giamarchi, E. Orignac, M. Rigol, One dimensional bosons: From condensed matter systems to ultracold gases. *Rev. Mod. Phys.* **83**, 1405–1466 (2011).

51. M. Girardeau, Relationship between systems of impenetrable bosons and fermions in one dimension. *J. Math. Phys.* **1**, 516–523 (1960).

52. J. C. Owens, M. G. Panetta, B. Saxberg, G. Roberts, S. Chakram, R. Ma, A. Vrajitoarea, J. Simon, D. I. Schuster, Chiral cavity quantum electrodynamics. *Nat. Phys.* **18**, 1048–1052 (2022).

53. F. Grusdt, N. Y. Yao, D. Abanin, M. Fleischhauer, E. Demler, Interferometric measurements of many-body topological invariants using mobile impurities. *Nat. Commun.* **7**, 11994 (2016).

54. N. Dupuis, R. Daviet, Bose-glass phase of a one-dimensional disordered Bose fluid: Metastable states, quantum tunneling, and droplets. *Phys. Rev. E.* **101**, 042139 (2020).

55. C. Meldgin, U. Ray, P. e. a. Russ, Probing the Bose glass superfluid transition using quantum quenches of disorder. *Nat. Phys.* **12**, 646–649 (2016).

56. J. Zhang, P. W. Hess, A. Kyprianidis, P. Becker, A. Lee, J. Smith, G. Pagano, I.-D. Potirniche, A. C. Potter, A. Vishwanath, N. Y. Yao, C. Monroe, Observation of a discrete time crystal. *Nature* **543**, 217–220 (2017).

57. S. Choi, J. Choi, R. Landig, G. Kucsko, H. Zhou, J. Isoya, F. Jelezko, S. Onoda, H. Sumiya, V. Khemani, C. von Keyserlingk, N. Y. Yao, E. Demler, M. D. Lukin, Observation of discrete time-crystalline order in a disordered dipolar many-body system. *Nature* **543**, 221–225 (2017).

58. C. S. Chiu, G. Ji, A. Mazurenko, D. Greif, M. Greiner, Quantum state engineering of a hubbard system with ultracold fermions. *Phys. Rev. Lett.* **120**, 243201 (2018).

59. J. Koch, T. M. Yu, J. Gambetta, A. A. Houck, D. I. Schuster, J. Majer, A. Blais, M. H. Devoret, S. M. Girvin, R. J. Schoelkopf, Charge-insensitive qubit design derived from the cooper pair box. *Phys. Rev. A* **76**, 042319 (2007).

60. F. Meinert, M. J. Mark, K. Lauber, A. J. Daley, H.-C. Nägerl, Floquet engineering of correlated tunneling in the Bose-Hubbard model with ultracold atoms. *Phys. Rev. Lett.* **116**, 205301e (2016).

61. M. A. Cazalilla, Differences between the tonks regimes in the continuum and on the lattice. *Phys. Rev. A* **70**, 041604 (2004).

62. A. Bijl, The lowest wave function of the symmetrical many particles system. *Phys. Ther.* **7**, 869–886 (1940).

63. M. A. Cazalilla, Bosonizing one-dimensional cold atomic gases. *J. Phys. B At. Mol. Opt. Phys.* **37**, S1 (2004).

64. C. N. Barrett, A. H. Karamlou, S. E. Muschinske, I. T. Rosen, J. Braumüller, R. Das, D. K. Kim, B. M. Niedzielski, M. Schuldt, K. Serniak, M. E. Schwartz, J. L. Yoder, T. P. Orlando, S. Gustavsson, J. A. Grover, W. D. Oliver, Learning-based calibration of flux crosstalk in transmon qubit arrays. *Phys. Rev. Applied* **20**, 024070 (2023).
